# Mesiotemporal Volume Loss Associated with Disorder Severity: A VBM Study in Borderline Personality Disorder

**DOI:** 10.1371/journal.pone.0083677

**Published:** 2013-12-18

**Authors:** Kirsten Labudda, Stefan Kreisel, Thomas Beblo, Markus Mertens, Oleg Kurlandchikov, Christian G. Bien, Martin Driessen, Friedrich G. Woermann

**Affiliations:** 1 Mara Hospital, Bethel Epilepsy Center, MRI Unit, Bielefeld, Germany; 2 Department of Psychiatry and Psychotherapy Bethel, Evangelisches Krankenhaus Bielefeld, Bielefeld, Germany; University of Medicine & Dentistry of NJ - New Jersey Medical School, United States of America

## Abstract

Results of MRI volumetry in Borderline Personality Disorder (BPD) are inconsistent. Some, but not all, studies reported decreased hippocampus, amygdala, and/or prefrontal volumes. In the current study, we used rater-independent voxel-based morphometry (VBM) in 33 female BPD patients and 33 healthy women. We measured gray matter (GM) volumes of the whole brain and of three volumes of interest (VOI), i.e., the hippocampus/parahippocampal gyrus, the amygdala and the anterior cingulate gyrus (ACC). Analyses were conducted using lifetime diagnoses of posttraumatic stress disorder (PTSD) and major depression (MD) as covariates. We used adversive childhood experiences and the numbers of BPD criteria (as an indicator of disorder severity) to investigate associations with GM volumes. We did not find volume differences between BPD patients and healthy subject, neither of the whole brain nor of the three VOIs, independent of presence or absence of comorbid PTSD and MD. We also did not find a relationship between childhood maltreatment and the patients’ brain volumes. However, within the patient group, the number of BPD criteria fulfilled was inversely correlated with left hippocampal/parahippocampal volume (x=-32, y=-23, z=-18, k=496, t=5.08, p=.007). Consequently, mesiotemporal GM volumes do not seem to differentiate patients from healthy subjects, but might be associated with symptom severity within the BPD group.

## Introduction

Borderline personality disorder (BPD) is a serious and chronic mental disorder characterized by affective instability and interpersonal disturbances. With a lifetime prevalence up to 6% it is a frequent personality disorder, especially in women. High rates of psychiatric comorbidities such as posttraumatic stress disorder (PTSD), major depression (MD), and substance use disorders have been reported in BPD patients [1]. The neuroanatomical substrate of BPD is poorly understood to date. Studies investigating regional brain volumes delivered inconsistent results, most likely due to small sample sizes, high (but mainly uncontrolled) rates of comorbidity, or heterogeneous patient populations, e.g. with respect to sex and age. The most frequent targets of those studies were the hippocampus and the amygdala. Some authors demonstrated severe volume losses of 10-34% within these regions, discussed to be associated with early traumatic experiences [2–4], [5–7]. Recently, Kuhlmann et al. [8] reported left-sided hippocampal volume reductions in 30 female BPD patients, but they did not replicate an association with early traumatic experiences. Results of Schmahl et al. [9] and Weniger et al. [5] suggested that mesiotemporal volume reductions cannot be entirely attributed to comorbid PTSD, a disorder that is associated with volume decreases in this region, too (see meta-analysis by Karl et al. [10]). Other authors did not find significant volume reductions in the amygdala and/or the hippocampus [11,12] or even reported volume increases within the amygdala [13]. Some studies found volume losses within frontal brain regions, such as the orbitofrontal cortex and the anterior cingulate gyrus (ACC) in BPD patients [4,12,14,15]. Goodman et al. [16] found smaller volumes of the ACC to be associated with more severe BPD symptoms in 13 BPD patients with comorbid MD. However, their analysis of the association between structural brain changes and symptom severity was restricted to this mesiofrontal brain region. 

In a group of BPD patients, childhood maltreatment experience has been reported to predict hypothalamus-pituitary-adrenal (HPA) dysfunctions [17]. Hippocampal volume reductions in BPD and PTSD patients have been discussed as a result of a traumatic stress induced HPA dysregulation. An association between childhood maltreatment and brain volume reductions of the hippocampus [18], but also of the insula and of mesial frontal brain areas [19] were recently reported in large normal population samples.

In the current study, we used voxel-based morphometry (VBM) to test our hypothesis of smaller volumes within the hippocampus, the amygdala and the ACC in a large sample of 33 female BPD patients. Additionally, we hypothesize that volume decreases within these predefined volumes of interest (VOI) are associated with BPD severity and the extent of childhood maltreatment. Using VBM enables us to explore further brain volume increases or decreases in the whole brain. Concurrent diagnoses of PTSD and/or MD were used as covariates in our analyses.

## Materials and Methods

### Subjects

All 33 BPD subjects were inpatients of the Department of Psychiatry and Psychotherapy Bethel, Ev. Hospital Bielefeld, Germany. Inclusion criterion was BPD diagnosis based on DSM-IV criteria assessment (SCID II). Exclusion criteria were current or lifetime anorexia, schizophrenia, schizoaffective disorder, major depressive disorder with psychotic symptoms, and alcohol or substance dependence within 6 months prior to study participation. We additionally did not include those patients and healthy subjects that could not be investigated with MRI, e.g. due to metal implants, claustrophobia or pregnancy. In addition to PTSD and MD (see Table 1), the following current axis I comorbid disorders were diagnosed within the patient group: panic disorder and/or agoraphobia (n=6), bulimia nervosa (n=5), past substance abuse (n=3), social phobia (n=2), obsessive compulsive disorder (n=2), generalized anxiety disorder (n=1), specific phobia (n=1), somatization disorder (n=1). Axis II comorbidities comprised of avoidant (n=6), obsessive compulsive (n=1), dependent (n=1), paranoid (n=1) and not otherwise specified personality disorders (n=9, depressive PD or passive aggressive PD). Sixteen of 33 patients had psychopharmacological treatment at the time of MRI scan: Nine patients were taking antidepressants only (6 patients with SSRIs, 2 patients with tricyclic antidepressants, 1 with a tetracyclic antidepressant). Three patients had neuroleptic medication only (perazone, quetiapine and promethazine), 1 patient took neuroleptic medication (olanzapine and promethazine) combined with the benzodiazepine lormetazepam. Three patients were treated with both, neuroleptic medication and antidepressants. One patient took antipsychotic (chlorprothixen), antiepileptic (valproic acid) and benzodiazepine (flunitrazepam) medication. 

**Table 1:** Characteristics of psychopathology in the BPD total group and of both patient subgroups.

	BPD whole group	Mild BPD	Severe BPD	statistics**^*1*^**
	(n=33)	(n=15)	(n=18)	
	mean (SD)	mean (SD)	mean (SD)	
Age (years)	30.51 (11.57)	29.47 (12.25)	31.39 (11.26)	t=-.47, p=.64
School education (years)	11.18 (1.49)	10.93 (1.39)	11.39 (1.58)	t=-.87, p=.39
Number of BPD criteria fulfilled	6.64 (1.14)	5.60 (0.51)	7.50 (0.71)	t=-8.70, p<.001
MD current	n=6	n=4	n=2	Chi^2^=1.33, p=.38
MD lifetime	n=23	n=10	n=13	Chi^2^=.12, p=.51
PTSD current	n=15	n=2	n=13	Chi^2^=11.44, p=.001
PTSD lifetime	n=17	n=4	n=13	Chi^2^=6.80, p=.02
Age of first trauma**^*2*^**	n=23 11.52 (10.36)	n=10 11.40 (6.88)	n=13 11.62 (5.28)	t=-.09, p=.93
CTQ sum score	69.52 (SD=19.43)	61.67 (SD=16.30)	76.06 (SD=19.81)	t=-2.25, p=.03
Number of neglect/abuse types experienced	3.61 (SD=1.05)	3.33 (SD=1.8)	3.83 (SD=1.25)	t=-.94, p=.36

^1^ subgroup comparison (for all t-Tests df=31, for all Chi^2^-Tests df =1)

^2^ for all patients who reported trauma (according to the DSM IV A criteria) independent of PTSD diagnosis (n=23, df=21)

Thirty-three healthy subjects were recruited via local newspaper advertisements and matched strictly according to age (BPD: mean=30.51 years, SD=11.57 vs. CG: mean=31.21 years, SD=11.03, t=0.25, df=64, p=.80) and school education (BPD: mean=11.18 years, SD=1.49 vs. CG: mean=11.30, SD=1.47, t=.33, df=64, p=.74). Subjects with current or past psychiatric disorders (according to SCID I and II assessment) were excluded. None of the healthy subjects took psychopharamcological medication or had a history of substance addiction.

### Ethics statement

The study’s procedure was in accordance with the Declaration of Helsinki and approved by the ethic committee of Muenster University, Germany. All subjects gave written informed consent prior to participation. In the consent form, subjects got detailed information concerning the study procedure. We also assured that the investigators are liable to medical confidentiality and that data will only be published anonymously. We also assured that subjects are free to decline study participation and that no treatment disadvantages will occur in the event of decline for the patients. All subjects agreed to the use of their data for scientific purposes.

### Assessment of current and lifetime psychopathology

All 33 patients and healthy subjects underwent psychopathological assessment using the German version of the Structural Clinical Interview for DSM IV for axis I and II diagnoses (SCID I and II). BPD severity was determined by the number of DSM IV criteria fulfilled (minimum=5, maximum=9), i.e. low severity was defined by 5 or 6 criteria fulfilled, and high severity by 7 and more criteria. Thirteen patients additionally completed the Borderline Symptom List (BSL, [20]). The BSL is a reliable self-report measure of borderline symptomatology based on the DSM-IV criteria for BPD. The 13 patients’ BSL score (mean total score=198.14, SD=90.11) was highly correlated with the number of BPD criteria fulfilled (*r*=.65, p=.016). Therefore, we used the number of criteria fulfilled (ranging from 5 to 9) as regressor representing BPD severity in our VBM analyses. The SCID I was used to diagnose current and lifetime posttraumatic stress disorder (PTSD) and major depression (MD). Psychopathological characteristics of the patients are summarized in Table 1.

The Childhood Trauma Questionnaire (CTQ, [21]) was administered to assess maltreatment during childhood. This 25-item questionnaire includes 5 scales to assess physical abuse, physical neglect, emotional abuse, emotional neglect and sexual abuse. We calculated the total sum score (maximum score=100) and additionally used the cutoff scores proposed by Walker et al. [22] to calculate the number of different maltreatment types experienced (maximum=5) in each patient.

### Voxel-based morphometry

A T1-weighted 3D sequence (MPRAGE, TR=11.1 ms, TE=4.3 ms, slice thickness 1.5 mm, FOV 230x230 mm, matrix 256x256) covering the whole brain was obtained from all subjects with an 1.5 Tesla Scanner (Siemens Magnetom Symphony, Erlangen, Germany).

The present study employed the VBM5 toolbox (Gaser, http://dbm.neuro.uni-jena.de/vbm) which utilizes the unified segmentation approach implemented in SPM5 (Ashburner and Friston, 2005 [23]). The images were resampled at a resolution of 1x1x1 mm, normalized to the SPM standard template, bias field corrected, and automatically segmented. The VBM5 toolbox extends the unified segmentation approach as it increases the quality of segmentation by applying a hidden Markov Field (HMRF) on the segmented tissue. This procedure removes isolated voxels that are unlikely to belong to the tissue class to which they have been assigned initially, thereby minimizing noise effects. The T1-weighted 3D-images of all subjects were segmented into GM, white matter and cerebrospinal fluid (CSF). The final tissue maps were modulated with the Jacobian determinants of the deformation parameters obtained from the normalization to the MNI standard space to correct voxel signal intensities for the amount of volume displacement during normalization. Modulation involves scaling by the amount of contraction, so that the total amount of GM in the modulated GM remains unchanged in comparison with the original images. The modulated images were corrected for non-linear warping. The resulting GM images were smoothed with a 12 mm FWHM Gaussian kernel and used for statistical whole brain comparison. Additionally, regions where there was an a priori hypothesis for GM changes were evaluated using a small volume correction within anatomically predefined volumes of interest (VOI) defined by using the WFU PickAtlas tool (Maldjian, http://www.nitrc.org/projects/wfu_pickatlas). 

Counts of supra-threshold active voxels and labeling of the anatomical clusters were performed using the template-based automated anatomical labeling tool for SPM [24] (Cyceron, http://www.cyceron.fr/index.php/en/plateforme-en/freeware).

**Figure 1:**
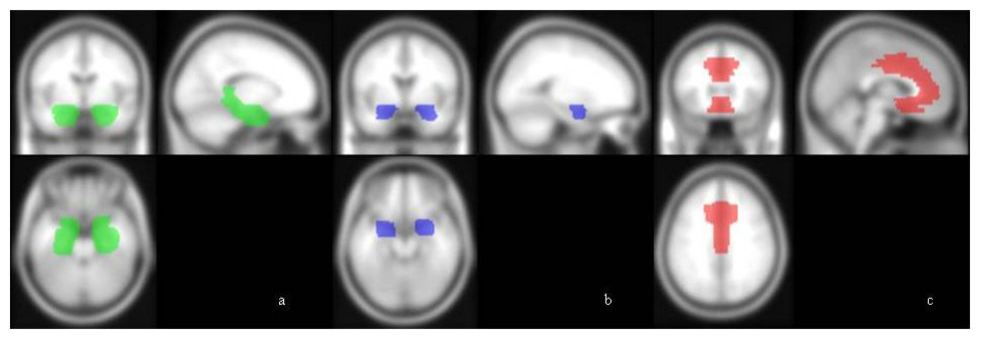
a-c Volumes of interest (VOI). All VOIs are anatomical templates provided by the WFU PickAtlas. Figure 1a shows the hippocampus/parahippocampal gyrus VOI, Figure 1b the amygdala VOI and Figure 1c the ACC VOI.

All statistical analyses have been performed using the default settings of SPM5 and the VBM5 toolbox. The main whole brain between-group comparison (CG>BPD and BPD>CG) were conducted at a conservative threshold (p<.05 FWE corrected) first and in order to not overlook more subtle changes repeated at a threshold of p<.001 uncorrected. The same stepwise procedure was used to test for volume changes in three VOIs: 1) hippocampus + parahippocampal gyrus; 2) amygdala and 2) anterior cingulate gyrus (ACC). The hippocampus + parahippocampal gyrus and the amygdala VOI are anatomical templates provided by the WFU PickAtlas based on the AAL Atlas. The ACC VOI comprises of BA 24, 25, 32 and 33 based on the Talairach Daemon database included in the WFU PickAtlas. All VOIs were dilated by 2 voxels using the 2D dilation function. VOIs are visualized in Figure 1a-c. 

All analyses were conducted without any covariates and additionally with PTSD lifetime (n=17) and MD lifetime (n=23, see Table 1) as covariates. We also calculated the group comparisons using medication (yes/no) as covariate, too (see Table S1). The number of BPD criteria fulfilled (see rationale above), the CTQ total sum score and the number of maltreatment types experienced were used as regressors in the VBM regression analyses. All analyses’ results are summarized in Table S1. Significant findings are described below.

## Results

### VBM: patients versus healthy subjects

Using both thresholds subsequently, we did not find significant GM differences when BPD patients and healthy subjects were compared in an unbiased whole brain analysis, neither with nor without MD, PTSD lifetime diagnoses or medication as covariates. We did not find significant group differences within the 3 VOIs (hippocampus/parahippocampal gyrus, amygdala, ACC), neither with nor without MD and PTSD lifetime diagnoses as covariates.

### VBM: severe BPD versus mild BPD

**Figure 2:**
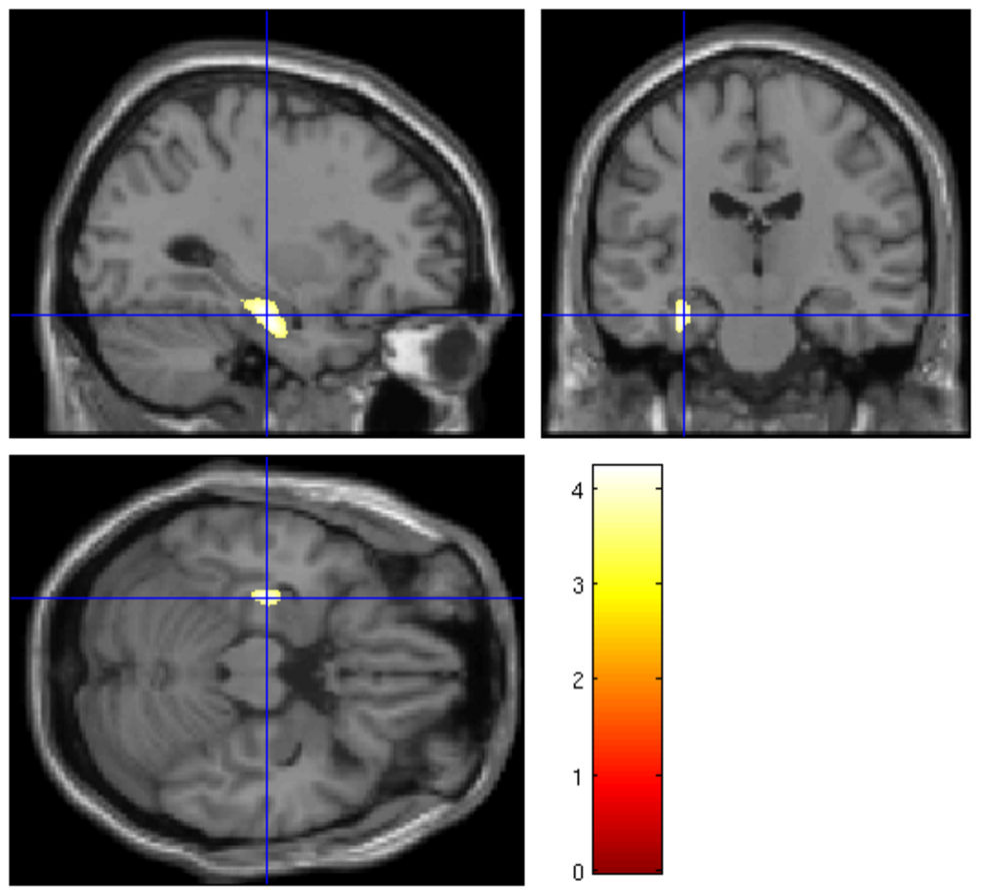
GM volume differences of the hippocampus. Significant difference of GM volume of the hippocampus/parahippocampal gyrus VOI in subjects with high versus low severity of BPD (cluster peak at x=-30, y=-18, z=-21, k=869, t=.4.22, p=.049 FWE-corrected, threshold of p<.001 uncorrected).

Patients with severe BPD symptoms (>7 BPD criteria, n=18) had significantly reduced volumes of the left (but not of the right) hippocampus/parahippocampal gyrus (x=-30, y=-18, z=-21, k=869, t=4.22, p=.049 corrected, VOI analysis with a threshold of p<.001 uncorrected) compared to those patients with mild BPD (5 or 6 BPD criteria, n=15, see Figure 2). The result of smaller hippocampus/parahippocampal gyrus volumes in patients with severe compared to patients with mild BPD remained significant when controlling for MD and PTSD lifetime diagnosis (t=3.96, p=.04 corrected) or medication (t=4.33, p=.04 corrected, see Table S1). In order to assure that the volume difference was not only due to a volume reduction of the parahippocampal gyrus in those patients with severe BPD, we calculated this analysis in the hippocampus alone, too. Results confirmed that the difference mainly occurred within the left hippocampus (x=-30, y=-18, z=-21, k=722, t=4.22, p=.031 corrected).

 We also compared both patient groups separately with the healthy subjects. We did not find any significant volume differences between the subgroups and healthy subjects, neither within the whole brain analyses, nor within the three VOI analyses.

### Correlations

**Figure 3:**
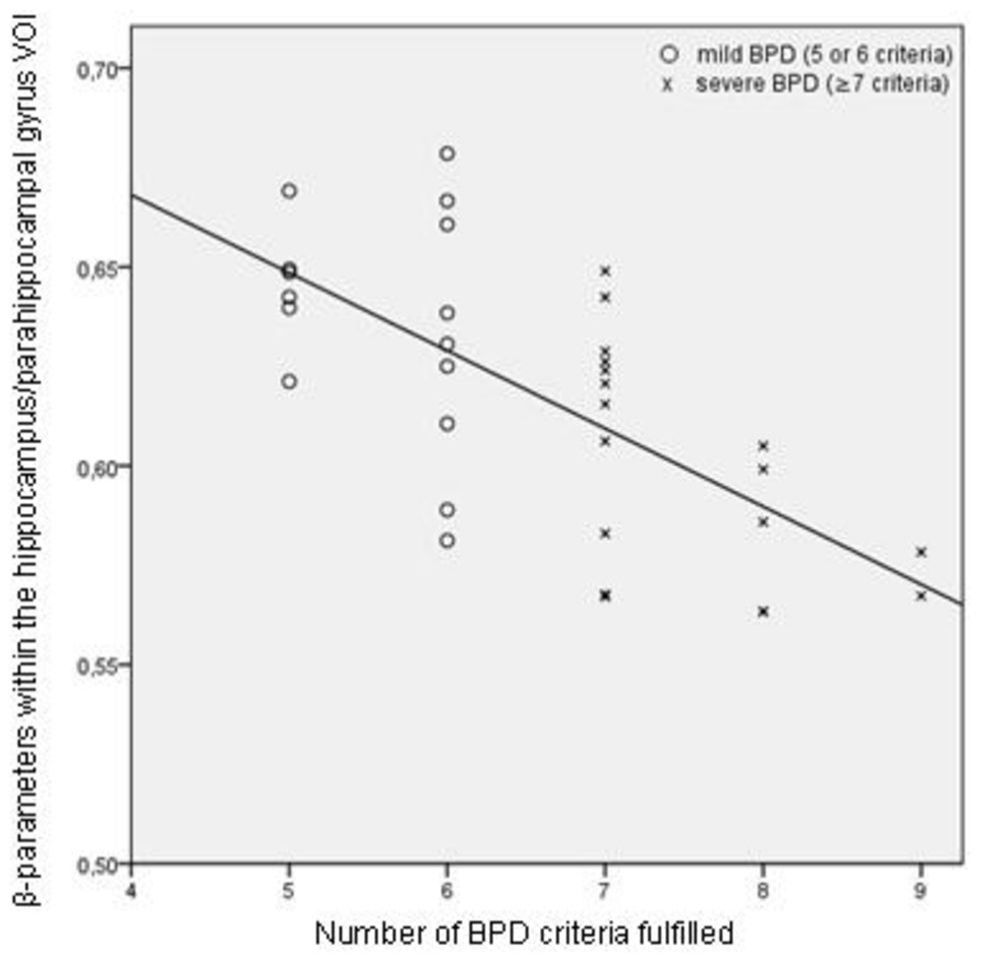
Correlation between hippocampus volume and BPD criteria. Correlation between the volume within left hippocampal region (β-parameters) and the number of BPD criteria fulfilled.

We found a significant inverse correlation between the VOI volume of the left (but not the right) hippocampus/parahippocampal gyrus and the number of BPD criteria fulfilled (threshold p<.05 FWE corrected: x=-32, y=-23, z=-18, k=707, t=5.08, p=.007) (Figure 3), i.e. smaller left hippocampal/parahippocampal volumes were associated with increasing number of BPD criteria fulfilled. This correlation remained significant at the corrected threshold when controlled for PTSD and MD lifetime diagnoses (k=403, t=4.87, p=.01 corrected). We did not find a correlation between GM volume and the number of BPD criteria fulfilled, neither in the amygdala and ACC VOI, nor in the whole brain analysis.

### Childhood maltreatment, trauma, PTSD and brain volumes

Neither the total CTQ scores, nor the number of abuse and/or neglect types during childhood was correlated significantly with brain volumes within the patient group (neither in the whole brain analyses, nor in the VOI analyses).

We also compared those patients with 4 or 5 reported maltreatment types (n=20) with those healthy subjects not reporting any maltreatment experience (n=19). In the patient group, we observed a trend towards smaller volumes of the right inferior frontal gyrus (x=55,y=20, z=15, k=562, t=4.42, p=.25 and x=46,y=9, z=32, k=624, t=4.41, p=.23 ), of the left occipital lobe (x=-39,y=-90, z=6, k=964, t=4.35, p=.14), and of the left postcentral gyrus (x=-64,y=10, z=-32, k=573, t=4.06, p=.25), but these differences failed to reach significance, even at the liberal threshold (p<.001 uncorrected).

We did not find a significant differences of the whole brain volumes and of the VOI volumes between BPD patients with (n=17) and without comorbid lifetime PTSD (n=16) and between these patient subgroups and the healthy subjects. Same accounts for BPD patients with (n=23) and without (n=10) trauma (DSM IV trauma A criterion fulfilled, independent of the presence or absence of PTSD): There were no significant volume differences between those patient subgroups. Both subgroups did not differ from the healthy subjects.

## Discussion

In the current study, we did not find significant GM differences between female patients with BPD and a group of healthy control subjects (even when using a liberal threshold), neither within the whole brain, nor within three anatomically defined VOIs comprising of the hippocampus/gyrus parahippocampalis, the amygdala, and the ACC. We also did not find associations between comorbid PTSD and MD diagnosis and the patients’ GM volumes. However, the number of BPD criteria fulfilled was inversely correlated with the volume of the left-sided hippocampal/parahippocampal VOI, i.e. greater BPD severity was associated with smaller volumes in this region.

Whereas hippocampal volume reductions have been reported in most studies with manually measured volumes [2,3,7,11,12,25], results of VBM studies are inconsistent: Our result of normal hippocampal volumes in BPD patients compared to control subjects is in line with most studies using VBM in BPD [13–15,26,27]. However, Kuhlmann et al. [8] and Soloff et al. [15] reported decreased bilateral hippocampal volumes in female BPD patients. Heterogenous results may be generally attributed to variables not controlled for in previous studies such as age, gender or psychiatric comorbidity. We only studied female patients closely matched for education and age with the control group and we analyzed the potential influence of the most frequent comorbidities, i.e. MD and PSTD. Comparing patients with control subjects, our results suggest no general brain volume differences independent of comorbid MD and PTSD. Soloff et al. [15] observed frontal and mesiotemporal volume decreases in BPD patients compared to healthy subjects. However, these differences diminished when impulsivity, a common characteristic in BPD, was used as covariate. Völlm et al. [28] also reported a negative association between impulsivity and GM volumes within frontal, anterior-temporal and parietal regions in BPD patients (those regions in which they found volume reductions in their BPD sample). Results of Zetzsche et al. [29], who showed an inverse correlation between hippocampal volumes and history of aggressive behaviour, also support the hypothesis that a lack of impulse control might be associated with hippocampal volumes in BPD patients. As our sample also included patients with a milder disorder severity, it is conceivable that impulsivity scores on average were lower in our patient sample. Although speculative, this might be the reason why we did not find any volume differences between our BPD patients and the comparison group.

With respect to amygdala volumes, our results of non-reduced amygdala volumes compared to healthy controls are in line with those of Kuhlmann et al. [8], Brunner et al. [14] and Völlm et al. [27]. However, Sorloff et al. [15] and Rusch et al. [26] reported decreased amygdala volumes in female BPD patients. Rusch at al. used a lower threshold in their VOI analyses (p=.001 uncorrected) and did not control for comorbid disorders (15 of 20 patients had a current PTSD diagnosis). Sorloff et al. showed that mesial temporal volume differences specifically occurred in female patients and diminished when controlling for impulsivity scores.

With respect to extra-temporal brain regions, some VBM studies reported decreased ACC volumes in BPD patients [13,14,27], others did not find differences in this brain region [8,26]. Goodman et al. [16] reported an inverse association between ACC volumes and severity of BPD. We did not find ACC volume decreases and/or any relationship between ACC volumes and BDP severity in our patient sample. Goodman et al. only included 13 subjects (male and female), all of them with comorbid MD. As shown by Sorloff et al. [15], using depression scores as covariate rendered ACC volume differences (previously only seen in male, but not female patients) between BPD patients and healthy subjects to be non-significant. Results of Brunner et al. [14] also suggested that ACC volume decreases are not BPD specific as BPD patients did not differ from a mixed psychiatric group in respect to ACC volumes. Thus, the MD comorbidity might have modified the association between ACC volumes and BPD severity in the study by Goodman and colleagues.

In contrast to recent results of Teicher et al. [18] and Dannlowski et al. [19] showing hippocampal (and extra-hippocampal) volume reductions in subjects from the normal population with childhood maltreatment experiences, we did not find maltreatment related brain abnormalities in BPD patients (neither in the whole brain nor in the VOI analyses). However, our results are in line with the studies by Driessen et al. [2], Schmahl et al. [9] and Kuhlmann et al. [8] who did not find a direct association between childhood maltreatment and manually measured hippocampal volumes (and of the amygdala and the ACC, see 8,9) in a female BPD group (but see also 11). These and the current study results indicate no direct link between traumatic experiences during childhood and brain morphology in BPD patients.

Altogether, methods of measuring morphological brain changes might have influenced results. Whereas hippocampal volume reductions have been reported in most studies with manually measured volumes [2,3,7,11,25] (but see also 12), only two [8,15] of six studies using VBM objectified hippocampal volume differences between healthy subject and BPD patients [13,14,26,27]. As VBM is based on normalized MRI images, it is sensitive to systematic shape differences resulting from misregistration in the spatial normalization step. We used the optimized VBM approach proposed by Gaser (http://dbm.neuro.uni-jena.de/vbm/) that includes a Hidden Markov Random Field model to reduce tissue misclassification and we further used a larger FWHM (12 mm) in order to further increase the accuracy of our VBM analysis (for a detailed discussion of methodological aspects of VBM such as potential normalization errors in VBM see e.g. 30–32). Measuring structural volumes manually is highly dependent on the qualification of the rater. It is further bias-prone, at least when the rater is not blinded to diagnoses. However, if raters are well trained and blinded, there is evidence that VBM and manual volumetry results in mesiotemporal and extratemporal regions widely correspond with each other [33–35]. Accordance between VBM results and those of other automated morphometric methods, such as cortical thickness measurement [36,37], also suggest that VBM is sensitive to reliably detect shape differences. Thus, in our study it is unlikely that the negative results were simply a methodological artifact. Recently, our group compared manually measured hippocampal volumes of 39 BPD patients and 39 matched healthy subjects (including our female patients and healthy subjects; [38]). In this study, we additionally analyzed volumes of hippocampal substructures (head, body and tail of the hippocampus). We did not find significant hippocampal volume reductions in the BPD patients compared to healthy subject - neither with respect to the total volume, nor with respect to the substructure volumes. Also in accordance with our VBM findings, the number of BPD criteria fulfilled was inversely correlated with the volume of the hippocampal head. This accordance further supports the assumption that the current results were not solely due to the method used. 

## Limitation

There is one clear limitation with our study: We used the BPD criteria to operationalize disorder severity. This is of limited validity as the number of criteria fulfilled did not provide information about the severity of each single symptom. Using established questionnaires to assess the severity of each symptom present would therefore be more precise and a preferable measure in future studies. However, we showed in a subgroup of our patient sample that the number of BPD criteria was highly correlated with BSL scores, a reliable measure of BPD severity. Therefore, we assume that the number of criteria represent a justifiable approximation of disorder severity. Nevertheless, the results have to be interpreted with caution.

## Summary

We did not find general differences of GM volumes between female patients with BPD and matched healthy subject, neither in a whole brain analysis, nor in mesiotemporal and frontal brain regions discussed as being reduced in BPD patients in some previous studies. Our results were independent of the most frequent comorbid mental disorders in BPD, i.e. MD and PTSD, and were also unlikely to be a methodological artefact. Within the patient group, the number of BPD was inversely correlated with left hippocampal/parahippocampal volumes. Thus, although not generally reduced in BPD patients, mesiotemporal GM volumes might be associated with symptom severity within BPD patient. We used the number of BPD criteria fulfilled as indicator for BPD severity, i.e. a higher number of BPD criteria is assumed to reflect more severe BPD. Although this interpretation seems to be conceivable in terms of representing a global severity estimation, further studies on this issue should assess symptom severity more detailed (e.g. by using questionnaires to measure the severity of BPD symptoms, separately). A more symptom-specific assessment of severity would allow to test whether or not the association between hippocampal volume and severity is rather general in nature or is explained by the intensity of some specific symptoms.

## Supporting Information

Table S1 **Results of all group comparisons and regression analyses** (thresholded at p<.001, ps presented were corrected on the cluster level). Given coordinates and anatomical notation refer to clusters’ peaks.”–“ indicates no suprathershold voxels in the respective comparison. All analyses were also calculated within the ACC and amygdala ROI, but there were no supratheshold voxels in any of these comparisons and regressions. We also did not find any supratheshold voxels within the positive correlation analyses. Significant results are indicated in bold font and are mentioned in the text.
(DOCX)
