# Supplementary material for: Mesiotemporal Volume Loss Associated with Disorder Severity: A VBM Study in Borderline Personality Disorder
Source: PLoS One. 2013 Dec 18;8(12):e83677. doi: 10.1371/journal.pone.0083677 (PMC3867453; doi:10.1371/journal.pone.0083677)
Supplement: Table S1 — Results of all group comparisons and regression analyses (thresholded at p<.001, ps presented were corrected on the cluster level). Given coordinates and anatomical notation refer to clusters’ peaks.”–“ indicates no suprathershold voxels in the respective comparison. All analyses were also calculated within the ACC and amygdala ROI, but there were no supratheshold voxels in any of these comparisons and regressions. We also did not find any supratheshold voxels within the positive correlation analyses. Significant results are indicated in bold font and are mentioned in the text. (DOCX) [file pone.0083677.s001.docx]

**Supporting Material**

**Table S1:** **Results of all group comparisons and regression analyses** (thresholded at p<.001, ps presented were corrected on the cluster level). Given coordinates and anatomical notation refer to clusters’ peaks.”–“ indicates no suprathershold voxels in the respective comparison. All analyses were also calculated within the ACC and amygdala ROI, but there were no supratheshold voxels in any of these comparisons and regressions. We also did not find any supratheshold voxels within the positive correlation analyses. Significant results are indicated in bold font and are mentioned in the text.

| Group comparisons | | |
| --- | --- | --- |
|  | Whole brain | Hippocampus/parahippocampal gyrus ROI |
| Healthy subjects > BPD, no covariates | - | - |
| BPD > healthy subjects, no covariates | - | - |
| BPD > healthy subjects, controlled for MD and PTSD | - | - |
| Healthy subjects > BPD, controlled for MD and PTSD | - | - |
| Healthy subjects > BPD, controlled for medication (yes/no) | Left posterior hippocampus: x=-30, y=-39, z=-3, k=412, t=3.67, p=.37; Right Middle frontal gyrus: x=41, y=12, z=29, k=259, t=3.63, p=.85; Right Posterior hippocampus: x=31, y=-35, z=-1, k=118, t=3.42, p=.92 | Left posterior hippocampus: x=-30, y=-39, z=-3, k=412, t=3.67, p=.10; Right posterior hippocampus: x=31, y=-35, z=-1, k=118, t=3.42, p=.16 |
| Severe BPD (n=18) vs. mild BPD (n=15) | - | - |
| Mild BPD > severe BPD | Left insula: x=-31, y=-6, z=15, k=461, t=4.27, p=.74; Left parahippocampal gyrus/hippocampus: x=-30, y=-18, z=-21, k=874, t=4.22, p=.52; Left middle temporal gyrus: x=-42, y=2, z=-33, k=275, t=3.68, p=.85 | **Left parahippocampal gyrus/hippocampus: x=-30, y=-18, z=-21, k=869, t=4.22, p=.04** |
| Severe BPD (n=18) vs. mild BPD (n=15), controlled for MD and PTSD | - | - |
| Mild BPD > severe BPD, controlled for MD and PTSD | Left middle temporal gyrus: x=-42, y=2, z=-33, k=307, t=4.32, p=.83; Left parahippocampal gyrus/hippocampus: x=-31, y=-17, z=-23, k=809, t=4.17, p=.55; Left insula: x=-31, y=-6, z=15, k=323, t=3.62, p=.82 | **Left parahippocampal gyrus/hippocampus: x=-31, y=-17, z=-23, k=355, t=3.96, p=.04** |
| Severe BPD (n=18) vs. mild BPD (n=15), controlled for medication (yes/no) | - | - |
| Mild BPD > severe BPD, controlled for medication (yes/no) | Left middle temporal gyrus: x=-42, y=2, z=-33, k=631, t=4.85, p=.65; Left insula: x=-31, y=-6, z=15, k=474, t=4.24, p=.74; Left parahippocampal gyrus/hippocampus: x=-30, y=-19, z=-19, k=973, t=3.78, p=.47; Left angular gyrus: x=-37, y=-64, z=29, k=144, t=3.80, p=.93 | **Left parahippocampal gyrus/hippocampus: x=-30, y=-19, z=-19, k=966, t=4.33, p=.04** |
| healthy subjects > severe BPD | Right inferior frontal gyrus: x=44, y=9, z=30, k=105, t=3.58, p=.39 | - |
| healthy subjects > mild BPD | - | Right posterior hippocampus: x=28, y=-37, z=2, k=42, t=3.46, p=.21 |
| Healthy subjects without maltreatment > BPD with maltreatment (4 or 5 types) | Right inferior frontal gyrus : x=55,y=20, z=15, k=562, t=4.42, p=.25; Right inferior frontal gyrus: x=46,y=9, z=32, k=624, t=4.41, p=.23; Left occipital lobe: x=-39,y=-90, z=6, k=964, t=4.35, p=.14); Left postcentral gyrus: x=-64,y=10, z=-32, k=573, t=4.06, p=.25 | - |
| BPD without PTSD > BPD without PTSD | Right occipital lobe: x=25, y=-87, z=12, k=190, t=3.82, p=.90; x=21, y=-98, z=-7, k=151, t=3.54, p=.98 | - |
| Healthy subjects > BPD without PTSD | - | - |
| Healthy subjects > BPD with PTSD | Right occipital lobe: x=29,y=-90, z=17, k=278, t=3.48, p=.84 | - |
| BPD without trauma > BPD with trauma | Right occipital lobe: x=23, y=-88, z=11, k=109, t=3.42, p=.93 | - |
| Healthy subjects > BPD without trauma | - | - |
| Healthy subjects > BPD with trauma | - | - |
| Regression analyses (negative contrast) | | |
| Number of BPD criteria | **Left parahippocampal gyrus/hippocampus: x=-32, y=-23, z=-18, k=2955, t=5.08, p=.02;** Left middle temporal gyrus: x=-43, y=3, z=-34, k=758, t=4.70, p=.58; Left insula: x=-35, y=3, z=18, k=125, t=3.46, p=.93; Right parahippocampal gyrus, hippocampus: x=31, y=-20, z=-21, k=121, t=3.30, p=.93 | **Left parahippocampal gyrus/hippocampus: x=-32, y=-23, z=-18, k=707, t=5.08, p=.007** |
| Number of BPD criteria, controlled for MD and PTSD | **Left parahippocampal gyrus/hippocampus: x=-32, y=-23, z=-18, k=2480, t=4.87, p=.03;** Left middle temporal gyrus: x=-42, y=3, z=-33, k=623, t=3.97, p=.65 | **Left parahippocampal gyrus/hippocampus: x=-32, y=-23, z=-18, k=403, t=4.87, p=.01** |
| Total CTQ score | Left medial frontal gyrus: x=-17, y=49, z=-1, k=205, t=4.51, p=.89; Left insula: x=-39, y=7, z=18, k=487, t=4.49, p=.73; Left occipital lobe: x=-13, y=-85, z=14, k=397, t=4.30, p=.79; Left precuneus: x=-17, y=-46, z=46, k=265, t=4.15, p=.86; Left middle temporal gyrus: x=-40, y=-73, z=8, k=263, t=4.11, p=.87; Right inferior frontal gyrus: x=54, y=21, z=14, k=122, t=3.81, p=.94; Right parahippocampal gyrus: x=27, y=-9, z=-31, k=181, t=3.71, p=.91; Left postcentral gyrus: x=-56, y=-17, z=33, k=191, t=3.62, p=.90 | Right parahippocampal gyrus: x=27, y=-9, z=-31, k=181, t=3.71, p=.52 |
| Number of CTQ abuse/neglect types | Left occipital lobe: x=-14, y=-84, z=14, k=259, t=4.18, p=.86; Right inferior frontal gyrus: x=46 y=30, z=3, k=210, t=3.50, p=.89;  x=54, y=21, z=16, k=170, t=3.88, p=.91 | - |
